# Compromised CD4:CD8 ratio recovery in people living with HIV aged over 50 years: an observational study

**DOI:** 10.1111/hiv.12800

**Published:** 2019-10-16

**Authors:** A Francis‐Morris, NE Mackie, J Eliahoo, F Ramzan, S Fidler, KM Pollock

**Affiliations:** ^1^ Section of Virology Department of Infectious Disease Imperial College London London UK; ^2^ Jefferiss Wing Imperial College Healthcare NHS Trust London UK; ^3^ Statistical Advisory Service Imperial College London London UK; ^4^ National Institute for Health Research Imperial Biomedical Research Centre London UK

**Keywords:** ageing, CD4 count, CD4:CD8 ratio, CD8 count, older people living with HIV, primary HIV infection

## Abstract

**Objectives:**

Persistent CD4:CD8 ratio inversion (< 1) is associated with mortality in older people. We investigated the interaction of the effects of baseline CD8 count and age at HIV diagnosis on CD4:CD8 ratio recovery with antiretroviral therapy (ART).

**Methods:**

An observational study (1 January 2007 to 31 December 2016) was carried out using routinely collected data from the HIV outpatient services at Imperial College Healthcare NHS Trust, London, UK. CD4 and CD8 counts, prior to and during ART, treatment during primary HIV infection (PHI) and HIV‐1 viral load were included in univariate and multivariate analyses using Cox proportional hazard regression.

**Results:**

Data were included for 876 patients starting ART, where HIV suppression was achieved. Of these patients, 741 of 876 (84.6%) were male and 507 of 876 (57.9%) were Caucasian. The median time on ART was 38 [interquartile range (IQR) 17–66] months. CD8 count change on ART was bidirectional; low CD8 counts (≤ 600 cells/μL) increased and high CD8 counts (> 900 cells/μL) decreased. The median pre‐ART CD4:CD8 ratio was 0.41 (IQR 0.24–0.63), and recovery (≥ 1) occurred in 274 of 876 patients (31.3%). Pre‐ and post‐ART CD4:CD8 ratios were lower in those aged > 50 years compared with young adults aged 18–30 years (*P *<* *0.001 and *P *=* *0.002, respectively). After adjustment, younger age at HIV diagnosis (*P *<* *0.001) and treatment during PHI (*P *<* *0.001) were favourable for CD4:CD8 ratio normalization.

**Conclusions:**

Older age (> 50 years) at HIV diagnosis was associated with persistent CD4:CD8 ratio inversion, whereas treatment of PHI was protective. These findings confirm the need for testing and early treatment of people aged > 50 years, and could be used in a risk management algorithm for enhanced surveillance.

## Introduction

Globally, the HIV epidemic is ageing; not only are people living with HIV (PLWH) growing older, but the incidence of HIV infection remains significant in people aged 50 years and over in the United States, and has not followed the decline observed in younger age groups in the UK 1, 2. The aged immune system loses functionality, rendering older people vulnerable to infectious disease and potentially cancer (reviewed in Foster *et al*. and Pera *et al*. 3, 4). The ageing process affects T‐cell subsets, particularly CD8 T‐cells 5. Loss of clonal diversity, increased CD8 count, and age‐related silencing of the interleukin (IL)‐7 receptor gene have been reported 5. These changes may be reflected in CD4:CD8 ratio inversion (< 1) in older people.

The impact of acute and chronic HIV infection on the immune system in older people is not fully understood. Despite advances in life expectancy, PLWH continue to be at higher risk of all‐cause mortality, and mortality from infectious and liver disease than the general population 6. HIV infection is associated with an increased prevalence of age‐related diseases, despite antiretroviral therapy (ART), and the prevalence of non‐AIDS‐related morbidity, such as cardiovascular disease, is higher than in the general population 7, 8. The mechanisms that drive this difference for PLWH on suppressive ART could be a consequence of persistent immune activation and inflammation 9. Whilst most PLWH are now successfully managed on ART by nonmedical staff, development of a clinical algorithm to select those at risk of complications for additional surveillance is now required to optimize care and resources.

Monitoring the CD4:CD8 ratio may serve such a purpose 10. The peripheral CD4 count has, for decades, been a reliable biomarker for monitoring immunosuppression and recovery with ART 11, 12. Widespread access to ART has greatly improved the prognosis in HIV infection, and, with continuous viral suppression, reduced the clinical need for CD4 count monitoring. Recovery of the CD4:CD8 ratio to normal levels (≥ 1) with ART is often incomplete, however, which could reflect chronic immune activation occurring in persistent viral infection, including cytomegalovirus (CMV) and HIV infections 13, 14, 15, 16. Treatment of HIV during early infection protects the CD4:CD8 ratio, improving recovery rates, but late diagnosis, when significant immune damage and viral exposure have occurred, is still commonplace 6, 16, 17.

Decline of the CD4:CD8 ratio occurs in the ageing general population. This has been associated with increased mortality rates in large Swedish studies and may be linked to CMV persistence 18, 19. Age‐related mechanisms underlying CD4:CD8 ratio decline include loss of thymic output, CD8 T‐cell oligoclonal outgrowth and accumulation of senescent T cells, including CD8 CD28^null^ T cells, which can be virally driven 20, 21. These changes contribute to an adaptive immune system that is less responsive and less flexible in older people. In PLWH, persistent CD4:CD8 ratio inversion can involve incomplete CD4 count recovery and/or persistently high CD8 count, and early initiation of ART supports normalization of the CD8 count 15, 22, 23. The HIV reservoir could be a driver for persistent CD4:CD8 ratio abnormality^24^. Given the central role CD8 T cells play in anticancer and antiviral immunity, the recovery of this T‐cell compartment is important for older people living with HIV. Identifying the associations with persistent CD4:CD8 ratio abnormality in PLWH despite ART may indicate selection criteria for those at risk of complications.

Previous studies have considered the association of CD4:CD8 ratio recovery in HIV‐1 infection with morbidity and mortality outcomes, but with conflicting results regarding the utility of this as a predictive biomarker in PLWH 25, 26. In the general population, the association of CD4:CD8 ratio inversion with excess mortality affecting older people indicates that persistent inversion might be of particular importance in older people diagnosed with or living with HIV infection 18, 27. We therefore investigated the impact of age at HIV diagnosis and baseline CD8 count on CD4:CD8 ratio recovery. Here we show that treatment during primary HIV infection (PHI) was associated with recovery of the CD4:CD8 ratio to ≥ 1. Conversely, even after adjustment, older age at HIV diagnosis had a negative impact on CD4:CD8 ratio recovery, despite CD4 count gains, particularly in those aged > 50 years.

## Methods

### Study design and setting

**Figure 1:**
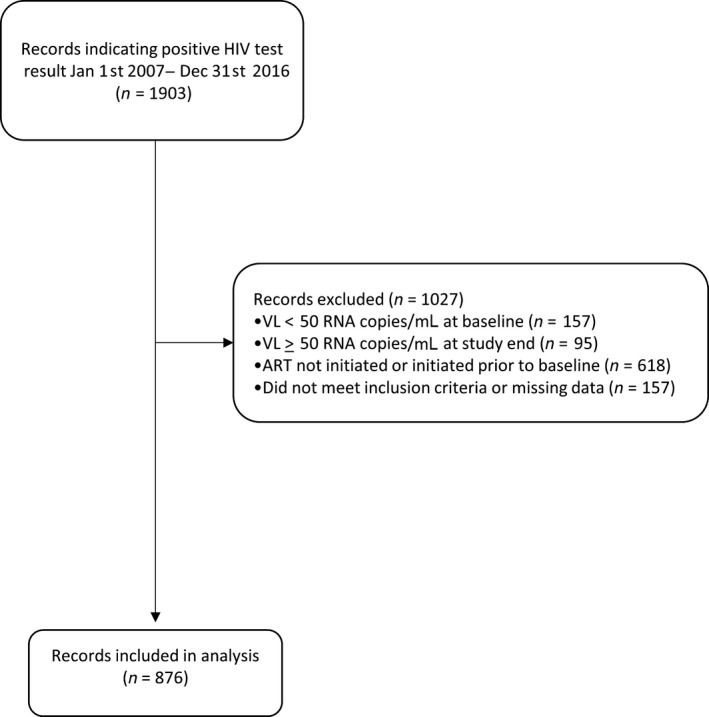
Flow diagram indicating selection of records for inclusion in analysis. ART, antiretroviral therapy; VL, viral load.

A retrospective study of PLWH starting ART during the 10‐year period from 1 January 2007 to 31st December 2016 was carried out, using routinely collected clinical data from the HIV out‐patient services at Imperial College Healthcare NHS Trust, London, UK. The flow diagram for data collection is shown in Figure 1.

#### Inclusion criteria

Data were collected from the electronic records system and anonymized prior to inclusion. All records of a first positive HIV test between the dates of 1 January 2007 and 31 December 2016 were included (*n *=* *1903). Tests for HIV infection using a third‐ or fourth‐generation assay were conducted in the Clinical Pathology Laboratory as part of routine diagnostic procedures. Cross‐linked anonymized database matching of data from the HIV Reservoir Targeting with Early Antiretroviral Therapy (HEATHER) study was undertaken to identify the records of those who had been diagnosed with primary HIV infection (PHI) and started on ART within 4 weeks [these patients were considered to have ‘treated PHI’ (tPHI)] 28. Entry criteria for the HEATHER study defined PHI as one or more of: positive HIV antibody test within 6 months of a negative test; negative HIV antibody test with positive polymerase chain reaction (PCR), protein p24 antigen or viral load; positive recent incident assay test algorithm consistent with recent infection; equivocal HIV antibody test with rising optical density on retest within 2 weeks; or symptomatic HIV seroconversion illness with positive antigen test and fewer than four bands in the western blot assay. Information on diagnosis and treatment of PHI is routinely available to members of the clinical care team.

#### Exclusion criteria

The following were excluded from the final data set: duplicate medical records, those with no last recorded clinic appointment, those with no record of starting ART, those for patients aged < 18 years, those for patients who acquired HIV infection through vertical transmission, those with fewer than two CD4:CD8 measurements, those with no record of ART initiation, and those where HIV viral load was < 50 HIV‐1 RNA copies/mL when ART was initiated. Those already on ART previously started elsewhere, or where there was exceptional natural viral control (elite controllers) were therefore excluded from the study. Records indicating failure to suppress HIV infection whilst on ART were excluded by identifying those with an HIV viral load ≥ 50 copies/ml at the end of the study period. All inclusion and exclusion criteria were applied to the raw data to generate the final data set on which all analyses were conducted.

Demographic data included age in years at the time of HIV testing, sex and ethnicity. CD4 and CD8 counts (cells/μL) and HIV viral load (copies/mL) measurements were performed by the accredited Clinical Pathology Laboratory using standard flow cytometry and molecular techniques. Measurements at baseline and all subsequent measurements during the study period were included. Categorical variables were selected after graphical visualization of the spread of the data. Baseline CD8 counts were categorized as low (0–350 cells/μL; CD8^Lo^), low‐normal (351–600 cells/μL; CD8^LoN^), high‐normal (601–900 cells/μL; CD8^HiN^), high (901–1500 cells/μL; CD8^Hi^) and very high (> 1500 cells/μL; CD8^VHi^) using reference ranges derived from the Clinical Pathology Laboratory performing the assays. Age at baseline was categorized into four groups, according to clinical relevance and spread of the data: 18–30 years (*n *=* *284); 31–40 years (*n *=* *306); 41–50 years (*n *=* *177); and > 50 years (*n *=* *109). The CD4:CD8 ratio and time on treatment during the study were derived mathematically. A normal CD4:CD8 ratio was defined as ≥ 1^19^.

### Statistical analysis

The anonymized data set was made available in microsoft excel format. The data were cleaned and analysed using excel 2013 and ibm spss statistics v24.0 software (IBM, New York, NY, USA). The primary outcome was the most recent CD4:CD8 ratio measurement in those on ART with suppressed HIV infection (viral load < 50 copies/mL). Quantitative values were assessed for normality using the Shapiro–Wilk test. In univariate analyses, paired data were investigated using the Wilcoxon signed‐rank test for nonparametric data. Analysis of inter‐group variance was performed with the Kruskal–Wallis test. Post hoc analyses were conducted using Dunn's test for multiple comparisons. Univariate analyses of continuous data were conducted using Spearman's rank correlation. HIV viral load was log_10_ transformed for univariate and multivariate analyses.

Multivariate analyses were conducted using the Cox proportional hazard regression model to estimate hazard ratios (HRs) with 95% confidence intervals (CIs) with duration of treatment as the timescale and persistent CD4:CD8 ratio inversion (< 1) as the outcome variable. Each participant accrued person‐time of follow‐up from the date of first starting HIV treatment until the last recorded CD4:CD8 ratio (the end of the study was 31 December 2016). Entry time was defined as the date of starting ART, and exit time was defined as the last recorded CD4:CD8 ratio prior to 1 January 2017. The multivariable model included age in years at date of diagnosis as a continuous variable, duration of ART, sex, baseline log_10_ HIV viral load (copies/mL), whether the patient was treated during PHI, ethnicity and baseline CD4 and CD8 counts (cells/μL).

## Results

### Cohort characteristics

Data from 876 records were analysed (Table 1). Of these patients, 741 of 876 (84.6%) were men, 507 of 876 (57.9%) were Caucasian, and 171 of 876 (19.5%) were black African or of other ethnicity. The median age at HIV diagnosis was 35 [interquartile range (IQR) 28–43] years, and 109 of 876 patients (12.4%) were aged > 50 years. At baseline, the median CD4:CD8 ratio was 0.41 (IQR 0.24–0.63) and the median HIV viral load was 34 000 (IQR 8500–10 300) copies/ml. The median time on ART was 38 (IQR 17–66) months. Recovery of CD4:CD8 ratio to ≥ 1 occurred in 274 of 876 cases (31.3%). CD4 count recovery to ≥ 500 cells/μL was achieved in 659 of 876 patients (75.2%); of these, 253 of 659 (38.4%) had a final CD4:CD8 ratio of ≥ 1. CD4 count recovery to ≥ 900 cells/μL was achieved in 164 of 876 patients (18.7%); of these, 90 of 164 (54.9%) had a final CD4:CD8 ratio of ≥ 1.

**Table 1:** Baseline characteristics

Category	Median (IQR)	Frequency (%)
Sex		
Male		741 (84.6)
Female		135 (15.4)
Ethnicity		
Caucasian		507 (57.9)
Black African/other		171 (19.5)
Mixed		58 (6.6)
Asian		60 (6.8)
Other		80 (9.1)
ART‐treated primary HIV infection		
Yes		75 (8.6)
No		801 (91.4)
Age at diagnosis (years)	35 (28–43)	
Baseline CD4 count (cells/μL)	390 (240–570)	
Baseline CD8 count (cells/μL)	890 (630–1250)	
Baseline CD4:CD8 ratio	0.41 (0.24–0.63)	
Pre‐ART HIV viral load (copies/mL)	34 000 (8500–10 300)	
Duration of ART (months)	38 (17‐66)	

ART, antiretroviral therapy; IQR, interquartile range.

### Association of high baseline CD8 count with persistent CD4:CD8 ratio inversion

In univariate analyses, across baseline (pre‐ART) CD8 count categories, baseline CD4 counts were lower in the CD8^Lo^ and CD8^LoN^ categories compared with the CD8^VHi^ category (*P *<* *0.001 and *P *<* *0.001, respectively; Table 2). Significant CD4 count gain was observed in all CD8 categories (Table 2). CD4 count gain was similar in the CD8^Lo^ and CD8^LoN^ groups [median 360 (IQR 170–460) and 330 (IQR 170–460) cells/μL, respectively; *P *=* *1.00]. These gains were greater than in the CD8^VHi^ group, where a median CD4 count gain of 190 (IQR 0–343) cells/μL was achieved (*P *=* *0.010 and *P *<* *0.001, respectively).

**Table 2:** Immune recovery by baseline CD8 count

CD8 category	Baseline CD4 count (cells/μL)		Final CD4 count (cells/μL)		Baseline CD8 count (cells/μL)	Final CD8 count (cells/μL)		Baseline CD4:CD8 ratio	Final CD4:CD8 ratio	
	Median (IQR)	*P*‐value*	Median (IQR)	*P*‐value†	Median (IQR)	Median (IQR)	*P*‐value‡	Median (IQR)	Median (IQR)	*P*‐value§
CD8^Lo^	80 (20–220)	< 0.001	470 (355–660)	< 0.001	280 (215–310)	510 (365–680)	< 0.001	0.32 (0.11–0.71)	0.86 (0.62–1.18)	< 0.001
CD8^LoN^	290 (140–450)	< 0.001	600 (450–813)	< 0.001	510 (440–560)	675 (498–873)	< 0.001	0.55 (0.30–0.88)	0.90 (0.70–1.28)	< 0.001
CD8^HiN^	400 (270–560)	0.029	650 (500–810)	< 0.001	760 (678–830)	760 (560–963)	0.165	0.55 (0.35–0.73)	0.86 (0.61–1.19)	< 0.001
CD8^Hi^	440 (280–610)	1.00	690 (540–895)	< 0.001	1130 (1020–1320)	890 (700–1150)	< 0.001	0.38 (0.25–0.55)	0.75 (0.59–1.00)	< 0.001
CD8^VHi^	485 (318–640)	Ref	650 (500–830)	< 0.001	1980 (1688–2563)	1065 (785–1485)	< 0.001	0.22 (0.14–0.32)	0.62 (0.44–0.88)	< 0.001

*P‐values are for pairwise analysis using the Kruskal–Wallis test with Dunn's post‐test comparison of baseline CD4 counts in each CD8 category using CD8^VHi^ as a reference category.

^†^ *P*‐values represent Wilcoxon signed‐rank test values for the baseline and final CD4 counts per baseline CD4 count category. Assessment of data distribution was conducted using the Shapiro–Wilk test. Difference values were calculated using the median (IQR) differences between baseline and final CD4 counts for each individual case.

^‡^ *P*‐values represent Wilcoxon signed‐rank test values for the baseline and final CD8 counts per baseline CD8 count category. Assessment of data distribution was conducted using the Shapiro–Wilk test. Difference values were calculated using the median (IQR) differences between baseline and final CD8 counts for each individual case.

^§^ *P*‐values represent Wilcoxon signed‐rank test values for the baseline and final CD4:CD8 ratio values in response to antiretroviral therapy per baseline CD8 count category. Assessment for data distribution was first conducted using the Shapiro–Wilk test. Difference values were calculated using the median (IQR) differences between baseline and final CD4:CD8 ratios for each individual case.

IQR, interquartile range; CD8^Lo^, low CD8 count; CD8^LoN^, low‐normal CD8 count; CD8^HiN^, high‐normal CD8 count; CD8^Hi^, high CD8 count; CD8^VHi^, very high CD8 count.

The direction of CD8 count change with ART was contingent upon baseline CD8 count. In the CD8^Lo^ and CD8^LoN^ groups, CD8 counts increased (*P *<* *0.001 and *P *<* *0.001, respectively; Table 2). Those in the CD8^HiN^ group had no significant change in CD8 count (*P *=* *0.165; Table 2). In the CD8^Hi^ and CD8^VHi^ groups (CD8 count > 900 cells/μL), ART reduced the CD8 count (*P *<* *0.001 and *P *<* *0.001; Table 2). Significant CD4:CD8 ratio gain was observed in all CD8 categories (*P *<* *0.001 for all CD8 categories) (Table 2). In pairwise analyses, those in the CD8^VHi^ category had a lower baseline and final CD4:CD8 ratio than those in any other CD8 category (Table S1). CD4:CD8 ratio recovery closest to optimal levels was observed in those in the CD8^LoN^ group, from a median of 0.55 (IQR 0.30–0.88) to 0.90 (IQR 0.70–1.28). The CD8^LoN^ group had the greatest frequency of CD4:CD8 ratio normalization (≥ 1); 67 of 166 (40.4%), compared with 13 of 37 (35.1%) in the CD8^Lo^, 97 of 250 (38.8%) in the CD8^HiN^, 74 of 293 (25.3%) in the CD8^Hi^ and 23 of 130 (17.7%) in the CD8^VHi^ category.

After adjustment, using the Cox proportional hazard regression model, a lower baseline CD4 count was associated with persistent CD4:CD8 ratio inversion (*P *<* *0.001; Table 3). Those with the lowest baseline CD4 count, 0–200 cells/μL, had the greatest risk of persistent CD4:CD8 ratio inversion (adjusted HR 15.104; 95% CI 10.108–22.568; *P *<* *0.001; Table 3). After adjustment, a higher baseline CD8 count was associated with persistent CD4:CD8 ratio inversion (*P *=* *0.025) and there was a trend for lower CD8 counts (CD8^Lo^, CD8^LoN^ and CD8^HiN^) to be associated with a lower risk of persistent CD4:CD8 ratio inversion (Table 3).

**Table 3:** Unadjusted and adjusted hazard ratios for persistent CD4:CD8 ratio inversion (< 1)

Category		uHR (95% CI)	*P*‐value*	aHR (95% CI)	*P*‐value†
Sex					
Female		0.833 (0.662–1.049)	0.120	0.855 (0.642–1.38)	0.283
Male		1*		1	
Age at diagnosis (years)		1.008 (1.000–1.015)	0.038	1.017 (1.009–1.024)	< 0.001
Ethnicity					0.756
Black African and black other		0.870 (0.715–1.058)	0.162	0.951 (0.778–1.163)	0.626
Caucasian		0.850 (0.668–1.081)	0.185	0.903 (0.687–1.188)	0.468
Other		1		1	
CD4 count at baseline					< 0.001
0–200 cells/μL		1.561 (1.142–2.134)	0.005	15.104 (10.108–22.568)	< 0.001
201–350 cells/μL		1.191 (0.869–1.647)	0.272	7.230 (5.068–10.313)	< 0.001
351–500 cells/μL		1.316 (0.961–1.802)	0.087	3.829 (2.744–5.342)	< 0.001
501–700 cells/μL		1.073 (0.775–1.484)	0.672	1.655 (1.190–2.303)	0.003
> 700 cells/μL		1		1	
CD8 count at baseline					0.025
0–350 cells/μL	CD8^Lo^	0.848 (0.544–1.321)	0.466	0.655 (0.414–1.070)	0.093
351–600 cells/μL	CD8^LoN^	0.760 (0.578–1.000)	0.050	0.764 (0.566–1.032)	0.079
601–900 cells/μL	CD8^HiN^	0.809 (0.631–1.038)	0.095	0.791 (0.609–1.027)	0.078
901–1500 cells/μL	CD8^Hi^	0.893 (0.708–1.125)	0.337	1.030 (0.806–1.316)	0.814
> 1500 cells/μL	CD8^Vhi^	1		1	
ART during primary HIV infection					
Yes		0.491 (0.334–0.721)	< 0.001	0.450 (0.302–0.671)	< 0.001
No		1		1	
Duration of ART		0.976 (0.973–0.978)	< 0.001	0.955 (0.951–0.958)	< 0.001
HIV viral load at baseline (log_10_ copies/mL)		1.120 (1.022–1.228)	0.016	1.245 (1.112–1.393)	< 0.001

*Redundant term denoted by 1.

^†^ *P*‐values represent the significance of the association with CD4:CD8 ratio reversal across the entire category using the Cox proportional hazards model.

aHR, adjusted hazard ratio; ART, antiretroviral therapy; CI, confidence interval; uHR, unadjusted hazard ratio.

### Nonlinear relationship of baseline CD8 count and viral load

Almost half the cohort, 423 of 876 patients (48.3%), had high (CD8^Hi^) or very high (CD8^VHi^) baseline CD8 counts. Previous work has indicated a potential association of AIDS mortality with high CD8 count 26. Given that unchecked HIV replication might drive a high CD8 count, we investigated the association of baseline CD8 count with viral load. A nonlinear relationship of baseline CD8 count with viral load was observed; those in the CD8^VHi^ and CD8^Lo^ categories had similarly high viral loads (*P * = 1.00; Table 4). Compared with CD8^VHi^, those in the CD8^LoN^, CD8^HiN^ and CD8^Hi^ categories had lower baseline HIV viral load (*P * = 0.001, *P * < 0.001 and *P * < 0.001, respectively; Table 4). HIV viral load correlated negatively with CD8 count in the lowest three categories (*r*_s_ = −0.159; *P * = 0.001) and positively with CD8 count in the highest two categories (*r*_s_ = 0.257; *P * < 0.001). After adjustment, a higher baseline HIV viral load was associated with persistent CD4:CD8 ratio inversion (adjusted HR 1.245; 95% CI 1.112–1.393; *P* < 0.001; Table 3).

**Table 4:** Viral load by baseline CD8 count

Baseline CD8 category	*n*	Baseline HIV VL (log_10_ copies/mL)	
		Median (IQR)	*P*‐value*
CD8^Lo^	37	5.00 (4.56–5.49)	1.000
CD8^LoN^	166	4.48 (3.90–5.03)	0.001
CD8^HiN^	250	4.38 (3.90–4.89)	< 0.001
CD8^Hi^	293	4.46 (3.79–4.92)	< 0.001
CD8^VHi^	130	4.90 (4.40–5.25)	Ref

*P‐values are for pairwise analysis using the Kruskal–Wallis test with Dunn's post‐test comparison of baseline HIV VL (log_10_ copies/mL) in each CD8 category using CD8^VHi^ as a reference category.

IQR, interquartile range; VL, viral load; CD8^Lo^, low CD8 count; CD8^LoN^, low‐normal CD8 count; CD8^HiN^, high‐normal CD8 count; CD8^Hi^, high CD8 count; CD8^VHi^, very high CD8 count.

### Association of older age at HIV diagnosis with CD4:CD8 ratio inversion

In univariate analyses, age at diagnosis correlated inversely with baseline CD4 count (*r*_s_ = −0.182; *P *<* *0.001) and CD4:CD8 ratio (*r*_s_ = −0.165; *P *<* *0.001) but not significantly with CD8 count (*r*_s_ = −0.065; *P *=* *0.055). CD4 count gain with ART was observed in all four age categories: 18–30, 31–40, 41–50 and > 50 years (*P *<* *0.001 for all age categories; Table 5). There was a small difference in CD4 count gain in those aged 31–40 years compared with those aged 18–30 years [median 210 (IQR 73–388) and 295 (IQR 110–470) cells/μL, respectively; *P *=* *0.014], but no differences between the other age groups. In those aged 18–30 years there was a significant CD8 count decrease with ART from a median of 930 (IQR 683–1328) cells/μL at baseline to 815 (IQR 612–1040) cells/μL (*P *<* *0.001; Table 5). There was also a significant CD8 count decrease with ART in those aged 31–40 years, from a median of 870 (IQR 618–1180) to 820 (IQR 618–1070) cells/μL (*P *=* *0.022; Table 5). Change in CD8 count with ART was not significant in those aged 41–50 and > 50 years (*P *=* *0.146 and *P *=* *0.135, respectively) (Table 5). CD8 count decrease with ART was therefore more likely at a younger age (≤ 40 years).

**Table 5:** Immune recovery by age at baseline

	Baseline CD4 count (cells/μL)	Final CD4 count (cells/μL)	*P*‐value*	Baseline CD8 count (cells/μL)	Final CD8 count (cells/μL)	*P*‐value*	Baseline CD4:CD8	Final CD4:CD8	Difference in CD4:CD8	*P*‐value*
Age at baseline (years)	Median (IQR)	Median (IQR)		Median (IQR)	Median (IQR)		Median (IQR)	Median (IQR)	Median (IQR)	
18–30	445 (320–600)	660 (533–848)	< 0.001	930 (683–1328)	815 (612–1040)	< 0.001	0.46 (0.31–0.65)	0.84 (0.66–1.14)	0.36 (0.17–0.61)	< 0.001
31–40	370 (218–553)	690 (518–840)	< 0.001	870 (618–1180)	820 (618–1070)	0.022	0.41 (0.24–0.63)	0.82 (0.58–1.12)	0.39 (0.14–0.66)	< 0.001
41–50	390 (200–595)	610 (470–830)	< 0.001	880 (570–1255)	770 (605–1220)	0.146	0.37 (0.21–0.70)	0.75 (0.51–1.09)	0.33 (0.12–0.58)	< 0.001
> 50	280 (135–495)	540 (415–785)	< 0.001	880 (575–1330)	830 (520–1150)	0.135	0.25 (0.14–0.55)	0.67 (0.47–1.02)	0.36 (0.16–0.54)	< 0.001

Assessment of data distribution was first conducted using the Shapiro–Wilk test. Difference values were calculated using the differences between baseline and final CD4: CD8 ratios for each individual.

*P‐values represent Wilcoxon signed‐rank test values for the baseline and final values in response to treatment for each age category.IQR, interquartile range.

In univariate analyses, significant CD4:CD8 ratio recovery with ART occurred in each age group (*P *<* *0.001 for each age category; Table 5). Those aged > 50 years had the lowest baseline median CD4:CD8 ratio, 0.25 (IQR 0.14–0.55) compared with 0.46 (IQR 0.31–0.65) for young adults aged 18–30 years (*P *<* *0.001; Tables 5 and S2). The final CD4:CD8 ratios in adults aged > 50 years were also lower than those in young adults aged 18–30 years [median 0.67 (IQR 0.47–1.02) versus 0.84 (IQR 0.66–1.14), respectively; *P *=* *0.002] (Tables 5 and S2). Increasing age at diagnosis was inversely correlated with final CD4:CD8 ratio (*r*_s_ = −0.138; *P *<* *0.001). In multivariate analyses, after adjustment and including those with tPHI, increasing age at diagnosis was associated with persistent CD4:CD8 ratio inversion (adjusted HR 1.017; 95% CI 1.009–1.024; *P *<* *0.001; Table 3).

### Impact of timing of ART on CD4:CD8 ratio recovery

In univariate analyses, there was an inverse association between CD4 count at baseline and duration of ART (*r*_s_ = −0.399; *P *<* *0.001), reflecting change in clinical practice over the past decade. There were 75 records where there had been a diagnosis of PHI and ART was started (tPHI). Of these patients, 70 of 75 (93.3%) were male, the median age was 31 (IQR 26–42) years and the median time from first positive HIV test was 1 (IQR 0–1) month. In those with tPHI, the median CD4 count was 530 (IQR 400–710) cells/μL, the median CD8 count was 1080 (IQR 820–1600) cells/μL, the median CD4:CD8 ratio was 0.53 (IQR 0.28–0.72) and the median HIV viral load was 4.88 (4.39–5.70) log_10_ copies/mL. Of the 423 patients who had CD8^Hi^ or CD8^VHi^ counts, 49 of 423 (11.6%) had tPHI. In multivariate analysis, where recent HIV seroconversion was identified and treated, this had a protective effect on the CD4:CD8 ratio (adjusted HR 0.450; 95% CI 0.302–0.671; *P *<* *0.001; Table 3). Increasing duration of ART was associated with a moderate risk reduction in persistent CD4:CD8 ratio inversion (adjusted HR 0.955; 95% CI 0.951–0.958; *P *<* *0.001; Table 3).

## Discussion

We present data from records of PLWH starting ART over one decade who achieved viral suppression, showing that older age at diagnosis was associated with suboptimal CD4:CD8 ratio recovery, particularly in those aged > 50 years. ART was beneficial for CD4:CD8 ratio normalization, but recovery to ≥ 1 occurred in only a third of patients (31.3%). Although CD4 count recovery was similar in younger and older age groups, CD8 count change did not occur in those diagnosed with HIV infection in the fifth decade or above. Those aged > 50 years had lower baseline and post‐ART CD4:CD8 ratios, and older age at diagnosis was associated with persistent CD4:CD8 ratio inversion even after adjustment and including those who started ART during PHI. These findings have implications for the long‐term health of PLWH into old age and for older people diagnosed with HIV infection.

With age, the immune system undergoes several maladaptive changes associated with physiological decline and the cumulative effect of chronic viral infection, especially CMV infection. Changes that affect adaptive immunity include loss of thymic output, reduced frequency of naïve cells and oligoclonal outgrowth of T cells and accumulation of CD8 CD28^null^ T cells 20, 21, 29. The immune risk profile, which encompasses several of these features, including CD4:CD8 ratio inversion, has been linked to increased mortality in older people 19. CD8 T cells are particularly vulnerable to epigenetic changes with age 5. Coupled with unfavourable changes in adaptive immunity over time in chronic HIV infection, as a consequence of immune activation, this may compound immunocompromise in older PLWH 30, 31.

We observed a wide range of baseline CD8 counts, with predominance in the high or very high range, reflecting the impact of HIV replication on cytotoxic T lymphocytes. The nonlinear relationship with viral load may explain why adjusting for viral load reduced the impact of baseline CD8 count on CD4:CD8 ratio recovery. Reduction in CD8 count with ART occurred in those with a baseline CD8 count > 900 cells/μL, which included those with a baseline CD4 count > 700 cells/μL. CD8 count reduction is therefore a feature of immune recovery even where CD4 count is not suppressed. CD8 counts are not usually stratified against clinical outcomes, but as part of the CD4:CD8 ratio might be useful in this regard^10^. The most favourable CD4:CD8 ratio recovery rate occurred in those with a baseline CD8 count of 351–600 cells/μL and normalization of the CD4:CD8 ratio was rare outside the range of 350–900 cells/μL.

Lower baseline CD4 counts were observed in older PLWH, which may reflect both immune decline and delay in diagnosis leading to greater immunosuppression prior to starting ART 2, 32. We demonstrated a powerful protective effect of early diagnosis and treatment of HIV infection in promoting immune recovery (CD4:CD8 ratio ≥ 1), in line with previous reports 16, 33. In the tPHI group, the median CD8 count was high [1080 (IQR 820–1600) cells/μL], but those with tPHI accounted for less than one‐fifth of all those with a baseline CD8 count in the > 900 cells/μL range. Treatment of PHI was therefore not solely accountable for the reduction in CD8 count with ART described. Initiation of ART, not just early treatment of HIV infection, could account for this effect. However, our data indicate that, whilst ART may be beneficial for CD4 count recovery, CD8 count and CD4:CD8 ratio normalization may be challenging in older PLWH.

A CD4:CD8 ratio of ≤ 1 was selected as the threshold for immune recovery in this study, reflecting previous work in HIV‐infected cohorts 16, 26. A CD4:CD8 ratio threshold of 1.2 has been used in some studies, and mean CD4:CD8 ratio may lie above this threshold, depending on the population in question 14, 34, 35. Evidence linking the CD4:CD8 ratio with undesirable immunological changes and poor clinical outcomes, including oxidative stress, cognitive disability and mortality, is associated with its inversion (CD4:CD8 ratio < 1) 18, 36, 37, 38. There are insufficient data to determine whether a difference of CD4:CD8 ratio recovery of 0.2 (between 1 and 1.2) is immunologically or clinically meaningful in PLWH.

### Limitations

We were unable to test for the effect of coinfecting pathogens, comorbidities or duration of viral suppression on CD4:CD8 ratio recovery, because of limitations in our data set. The presence of coinfecting pathogens, such as CMV, adversely affects the CD4:CD8 ratio 39, 40, 41. Teasing out the difference between HIV‐ and CMV‐mediated effects requires a large cohort, as the majority of PLWH are CMV‐antibody positive 39. Data from such studies, whilst of mechanistic significance, have limited clinical application in the absence of a safe and scalable vaccine or treatment that could meaningfully alter outcomes related to CMV persistence. The effects of other viruses, including hepatitis C virus, are more immediately modifiable with treatment 42, but data were insufficient to investigate this in the present study. Finally, although we showed relationships of age at diagnosis and baseline CD8 count with immune recovery we did not have data on subsequent clinical outcomes.

### Concluding remarks

The incidence and prevalence of HIV infection are significant in older people, in whom immunity is vulnerable to both age‐related decline and unfavourable adaptation to chronic infection. In this study, treatment during PHI was protective, but CD4:CD8 ratio recovery was nonetheless suboptimal in those aged >50 years. These data indicate that targeted testing and treatment of older people should be a priority for PLWH, alongside enhanced surveillance where the CD4:CD8 ratio fails to normalize.

## Ethical review

Data from electronic clinical records without patient identifiers were made available to the clinical care team on request and anonymized prior to analysis in accordance with National Research Ethics Service (NRES) guidance on using previously collected clinical data for research purposes (http://www.hra.nhs.uk/documents/2013/09/does-my-project-require-rec-review.pdf). The HEATHER study was approved by the NRES Committee West Midlands ‐ South Birmingham (ref. 14/WM/1104).

## Supporting information


**Table S1.** Pairwise analysis of difference in baseline and final CD4:CD8 ratios by CD8 category

**Table S2.** Pairwise analysis of age at baseline and difference in baseline and final CD4:CD8 ratios

## References

[1] PHE . HIV:annual data tables; 2018 Available at https://www.gov.uk/government/statistics/hiv-annual-data-tables.

[2] CDC . HIV Among People Aged 50 and Over; 2018 Available at https://www.cdc.gov/hiv/group/age/olderamericans/index.html.

[3] Foster AD , Sivarapatna A , Gress RE . The aging immune system and its relationship with cancer. Aging Health 2011; 7: 707–718. 22121388 10.2217/ahe.11.56 PMC3222953

[4] Pera A , Campos C , Lopez N *et al* Immunosenescence: implications for response to infection and vaccination in older people. Maturitas 2015; 82: 50–55. 26044074 10.1016/j.maturitas.2015.05.004

[5] Ucar D , Marquez EJ , Chung CH *et al* The chromatin accessibility signature of human immune aging stems from CD8(+) T cells. J Exp Med 2017; 214: 3123–3144. 28904110 10.1084/jem.20170416 PMC5626401

[6] Croxford S , Kitching A , Desai S *et al* Mortality and causes of death in people diagnosed with HIV in the era of highly active antiretroviral therapy compared with the general population: an analysis of a national observational cohort. Lancet Public Health 2017; 2: e35–e46. 29249478 10.1016/S2468-2667(16)30020-2

[7] Guaraldi G , Orlando G , Zona S *et al* Premature age‐related comorbidities among HIV‐infected persons compared with the general population. Clin Infect Dis 2011; 53: 1120–1126. 21998278 10.1093/cid/cir627

[8] Rasmussen LD , May MT , Kronborg G *et al* Time trends for risk of severe age‐related diseases in individuals with and without HIV infection in Denmark: a nationwide population‐based cohort study. Lancet HIV 2015; 2: e288–e298. 26423253 10.1016/S2352-3018(15)00077-6

[9] Wada NI , Jacobson LP , Margolick JB *et al* The effect of HAART‐induced HIV suppression on circulating markers of inflammation and immune activation. AIDS 2015; 29: 463–471. 25630041 10.1097/QAD.0000000000000545 PMC4311407

[10] Serrano‐Villar S , Deeks SG . CD4/CD8 ratio: an emerging biomarker for HIV. Lancet HIV 2015; 2: e76–e77. 26424546 10.1016/S2352-3018(15)00018-1

[11] Taylor JM , Fahey JL , Detels R , Giorgi JV . CD4 percentage, CD4 number, and CD4:CD8 ratio in HIV infection: which to choose and how to use. J Acquir Immune Defic Syndr 1989; 2: 114–124. 2495346

[12] Le T , Wright EJ , Smith DM *et al* Enhanced CD4+ T‐cell recovery with earlier HIV‐1 antiretroviral therapy. N Engl J Med 2013; 368: 218–230. 23323898 10.1056/NEJMoa1110187 PMC3657555

[13] Castilho JL , Shepherd BE , Koethe J *et al* CD4+/CD8+ ratio, age, and risk of serious noncommunicable diseases in HIV‐infected adults on antiretroviral therapy. AIDS 2016; 30: 899–908. 26959354 10.1097/QAD.0000000000001005 PMC4785819

[14] Leung V , Gillis J , Raboud J *et al* Predictors of CD4:CD8 ratio normalization and its effect on health outcomes in the era of combination antiretroviral therapy. PLoS ONE 2013; 8: e77665. 24204912 10.1371/journal.pone.0077665 PMC3813720

[15] Winston A , Jose S , Fisher M *et al* Host, disease, and antiretroviral factors are associated with normalization of the CD4:CD8 ratio after initiating antiretroviral therapy. J Allergy Clin Immunol 2015; 136: 1682–1685.e1. 26253341 10.1016/j.jaci.2015.05.047

[16] Thornhill J , Inshaw J , Oomeer S *et al* Enhanced normalisation of CD4/CD8 ratio with early antiretroviral therapy in primary HIV infection. J Int AIDS Soc 2014; 17: 19480. 25393989 10.7448/IAS.17.4.19480 PMC4224908

[17] Seng R , Goujard C , Krastinova E *et al* Influence of lifelong cumulative HIV viremia on long‐term recovery of CD4+ cell count and CD4+/CD8+ ratio among patients on combination antiretroviral therapy. AIDS 2015; 29: 595–607. 25715104 10.1097/QAD.0000000000000571

[18] Wikby A , Mansson IA , Johansson B , Strindhall J , Nilsson SE . The immune risk profile is associated with age and gender: findings from three Swedish population studies of individuals 20‐100 years of age. Biogerontology 2008; 9: 299–308. 18369735 10.1007/s10522-008-9138-6

[19] Strindhall J , Skog M , Ernerudh J *et al* The inverted CD4/CD8 ratio and associated parameters in 66‐year‐old individuals: the Swedish HEXA immune study. Age (Dordr) 2013; 35: 985–991. 22415616 10.1007/s11357-012-9400-3 PMC3636392

[20] Hadrup SR , Strindhall J , Kollgaard T *et al* Longitudinal studies of clonally expanded CD8 T cells reveal a repertoire shrinkage predicting mortality and an increased number of dysfunctional cytomegalovirus‐specific T cells in the very elderly. J Immunol 2006; 176: 2645–2653. 16456027 10.4049/jimmunol.176.4.2645

[21] Gui J , Mustachio LM , Su DM , Craig RW . Thymus size and age‐related thymic involution: early programming, sexual dimorphism, progenitors and stroma. Aging Dis 2012; 3: 280–290. 22724086 PMC3375084

[22] Cao W , Mehraj V , Kaufmann DE , Li T , Routy JP . Elevation and persistence of CD8 T‐cells in HIV infection: the Achilles heel in the ART era. J Int AIDS Soc 2016; 19: 20697. 26945343 10.7448/IAS.19.1.20697 PMC4779330

[23] Cao W , Mehraj V , Trottier B *et al* Early initiation rather than prolonged duration of antiretroviral therapy in HIV infection contributes to the normalization of CD8 T‐cell counts. Clin Infect Dis 2016; 62: 250–257. 26349551 10.1093/cid/civ809 PMC4690481

[24] Chun TW , Justement JS , Pandya P *et al* Relationship between the size of the human immunodeficiency virus type 1 (HIV‐1) reservoir in peripheral blood CD4+ T cells and CD4+:CD8+ T cell ratios in aviremic HIV‐1‐infected individuals receiving long‐term highly active antiretroviral therapy. J Infect Dis 2002; 185: 1672–1676. 12023777 10.1086/340521

[25] Serrano‐Villar S , Sainz T , Lee SA *et al* HIV‐infected individuals with low CD4/CD8 ratio despite effective antiretroviral therapy exhibit altered T cell subsets, heightened CD8+ T cell activation, and increased risk of non‐AIDS morbidity and mortality. PLoS Pathog 2014; 10: e1004078. 24831517 10.1371/journal.ppat.1004078 PMC4022662

[26] Trickey A , May MT , Schommers P *et al* CD4:CD8 ratio and CD8 count as prognostic markers for mortality in human immunodeficiency virus‐infected patients on antiretroviral therapy: the antiretroviral therapy cohort collaboration (ART‐CC). Clin Infect Dis 2017; 65: 959–966. 28903507 10.1093/cid/cix466 PMC5850630

[27] Huppert FA , Pinto EM , Morgan K , Brayne C . Survival in a population sample is predicted by proportions of lymphocyte subsets. Mech Ageing Dev 2003; 124: 449–451. 12714252 10.1016/s0047-6374(03)00021-6

[28] NHS . HEATHER ‐ Health Research Authority. Available at https://www.hra.nhs.uk/planning-and-improving-research/application-summaries/research-summaries/heather/.

[29] Qin L , Jing X , Qiu Z *et al* Aging of immune system: Immune signature from peripheral blood lymphocyte subsets in 1068 healthy adults. Aging (Albany NY) 2016; 8: 848–859. 26886066 10.18632/aging.100894 PMC4931839

[30] Cockerham LR , Siliciano JD , Sinclair E *et al* CD4 + and CD8 + T cell activation are associated with HIV DNA in resting CD4 + T cells. PLoS ONE 2014; 9: e110731. 25340755 10.1371/journal.pone.0110731 PMC4207702

[31] Hatano H , Jain V , Hunt PW *et al* Cell‐based measures of viral persistence are associated with immune activation and programmed cell death protein 1 (PD‐1)‐expressing CD4 + T cells. J Infect Dis 2013; 208: 50–56. 23089590 10.1093/infdis/jis630 PMC3666131

[32] Kilpatrick RD , Rickabaugh T , Hultin LE *et al* Homeostasis of the naive CD4+ T cell compartment during aging. J Immunol 2008; 180: 1499–1507. 18209045 10.4049/jimmunol.180.3.1499 PMC2940825

[33] Jain V , Hartogensis W , Bacchetti P *et al* Antiretroviral therapy initiated within 6 months of HIV infection is associated with lower T‐cell activation and smaller HIV reservoir size. J Infect Dis 2013; 208: 1202–1211. 23852127 10.1093/infdis/jit311 PMC3778965

[34] Saeed Z , Rowan A , Greiller C , Taylor GP , Pollock KM . Enhanced T‐cell maturation and monocyte aggregation are features of cellular inflammation in human T‐lymphotropic virus type‐1‐associated myelopathy. Clin Infect Dis 2019 10.1093/cid/ciz369 31063543

[35] McBride JA , Striker R . Imbalance in the game of T cells: what can the CD4/CD8 T‐cell ratio tell us about HIV and health? PLoS Pathog 2017; 13: e1006624. 29095912 10.1371/journal.ppat.1006624 PMC5667733

[36] Luz Correa B , Ornaghi AP , Cerutti Muller G *et al* The inverted CD4:CD8 ratio is associated with cytomegalovirus, poor cognitive and functional states in older adults. NeuroImmunoModulation 2014; 21: 206–212. 24504177 10.1159/000356827

[37] Olsson J , Wikby A , Johansson B , Lofgren S , Nilsson BO , Ferguson FG . Age‐related change in peripheral blood T‐lymphocyte subpopulations and cytomegalovirus infection in the very old: the Swedish longitudinal OCTO immune study. Mech Ageing Dev 2000; 121: 187–201. 11164473 10.1016/s0047-6374(00)00210-4

[38] Muller GC , Gottlieb MG , Luz Correa B , Filho IG , Moresco RN , Bauer ME . The inverted CD4:CD8 ratio is associated with gender‐related changes in oxidative stress during aging. Cell Immunol 2015; 296: 149–154. 26051633 10.1016/j.cellimm.2015.05.006

[39] Poizot‐Martin I , Allavena C , Duvivier C *et al* CMV+ serostatus associates negatively with CD4:CD8 ratio normalization in controlled hiv‐infected patients on cART. PLoS ONE 2016; 11: e0165774. 27824907 10.1371/journal.pone.0165774 PMC5100980

[40] Freeman ML , Mudd JC , Shive CL *et al* CD8 T‐cell expansion and inflammation linked to CMV coinfection in ART‐treated HIV infection. Clin Infect Dis 2016; 62: 392–396. 26400999 10.1093/cid/civ840 PMC4706630

[41] Barrett L , Stapleton SN , Fudge NJ , Grant MD . Immune resilience in HIV‐infected individuals seronegative for cytomegalovirus. AIDS 2014; 28: 2045–2049. 25265072 10.1097/QAD.0000000000000405

[42] Kuniholm MH , O'Brien TR , Prokunina‐Olsson L *et al* Association of hepatitis C virus infection With CD4/CD8 Ratio in HIV‐positive women. J Acquir Immune Defic Syndr 2016; 72: 162–170. 27183178 10.1097/QAI.0000000000000928 PMC4874499

