# Supplementary material for: Compromised CD4:CD8 ratio recovery in people living with HIV aged over 50 years: an observational study
Source: HIV Med. 2019 Oct 16;21(2):109–18. doi: 10.1111/hiv.12800 (PMC7003811; doi:10.1111/hiv.12800)
Supplement: Supplementary file 1 — Table S1. Pairwise analysis of difference in baseline and final CD4:CD8 ratios by CD8 category [file HIV-21-109-s001.docx]

**Supplementary table 1. Pairwise analysis of difference in baseline and final CD4:CD8 ratio by CD8 category**

| **Baseline CD4:CD8 ratio** | **CD8^Lo^** | **CD8^LoN^** | **CD8^HiN^** | **CD8^Hi^** | **CD8^VHi^** |
| --- | --- | --- | --- | --- | --- |
| **CD8^Lo^** | NA | NA | NA | NA | NA |
| **CD8^LoN^** | 0.53 | NA | NA | NA | NA |
| **CD8^HiN^** | 0.018 | 1.000 | NA | NA | NA |
| **CD8^Hi^** | 1.000 | <0.001 | <0.001 | NA | NA |
| **CD8^VHi^** | 0.005 | <0.001 | <0.001 | 0.001 | NA |
| **Final CD4:CD8 ratio** |  |  |  |  |  |
| **CD8^Lo^** | NA | NA | NA | NA | NA |
| **CD8^LoN^** | 1.00 | NA | NA | NA | NA |
| **CD8^HiN^** | 1.00 | 1.00 | NA | NA | NA |
| **CD8^Hi^** | 1.00 | 0.002 | 0.117 | NA | NA |
| **CD8^VHi^** | 0.012 | <0.001 | <0.001 | 0.001 | NA |

p values represent differences between baseline and final CD4:CD8 ratio values in each CD8 category using Kruskal-Wallis test with Dunn’s post test analysis
