# Supplementary material for: Compromised CD4:CD8 ratio recovery in people living with HIV aged over 50 years: an observational study
Source: HIV Med. 2019 Oct 16;21(2):109–18. doi: 10.1111/hiv.12800 (PMC7003811; doi:10.1111/hiv.12800)
Supplement: Supplementary file 2 — Table S2. Pairwise analysis of age at baseline and difference in baseline and final CD4:CD8 ratios [file HIV-21-109-s002.docx]

**Supplementary Table 2. Pairwise analysis of age at baseline and difference in baseline and final CD4:CD8 ratios**

| **Baseline CD4:CD8 ratio** | **18-30ys** | **31-40ys** | **41-50ys** | **>50ys** |
| --- | --- | --- | --- | --- |
| **18-30ys** | NA | NA | NA | NA |
| **31-40ys** | 0.137 | NA | NA | NA |
| **41-50ys** | 0.171 | 1.000 | NA | NA |
| **>50ys** | <0.001 | 0.002 | 0.012 | NA |
| **Final CD4:CD8 ratio** | **18-30ys** | **31-40ys** | **41-50ys** | **>50ys** |
| **18-30ys** | NA | NA | NA | NA |
| **31-40ys** | 1.000 | NA | NA | NA |
| **41-50ys** | 0.088 | 1.000 | NA | NA |
| **>50ys** | 0.002 | 0.056 | 1.000 | NA |

p values represent differences between baseline and final CD4:CD8 ratio values in each age category using Kruskal-Wallis test with Dunn’s post test analysis
